# When Place Matters: Shuttling of Enolase-1 Across Cellular Compartments

**DOI:** 10.3389/fcell.2019.00061

**Published:** 2019-04-26

**Authors:** Miroslava Didiasova, Liliana Schaefer, Malgorzata Wygrecka

**Affiliations:** ^1^Department of Biochemistry, Faculty of Medicine, Universities of Giessen and Marburg Lung Center, Giessen, Germany; ^2^Institute of Pharmacology and Toxicology, Goethe University Frankfurt, Frankfurt, Germany; ^3^Member of the German Center for Lung Research, Giessen, Germany

**Keywords:** enolase-1, multitasking protein, glycolysis, protein compartmentalization, cancer

## Abstract

Enolase is a glycolytic enzyme, which catalyzes the inter-conversion of 2-phosphoglycerate to phosphoenolpyruvate. Altered expression of this enzyme is frequently observed in cancer and accounts for the Warburg effect, an adaptive response of tumor cells to hypoxia. In addition to its catalytic function, ENO-1 exhibits other activities, which strongly depend on its cellular and extracellular localization. For example, the association of ENO-1 with mitochondria membrane was found to be important for the stability of the mitochondrial membrane, and ENO-1 sequestration on the cell surface was crucial for plasmin-mediated pericellular proteolysis. The latter activity of ENO-1 enables many pathogens but also immune and cancer cells to invade the tissue, leading further to infection, inflammation or metastasis formation. The ability of ENO-1 to conduct so many diverse processes is reflected by its contribution to a high number of pathologies, including type 2 diabetes, cardiovascular hypertrophy, fungal and bacterial infections, cancer, systemic lupus erythematosus, hepatic fibrosis, Alzheimer’s disease, rheumatoid arthritis, and systemic sclerosis. These unexpected non-catalytic functions of ENO-1 and their contributions to diseases are the subjects of this review.

## Introduction

Enolase is a metalloenzyme that catalyzes the inter-conversion of 2-phosphoglycerate to phosphoenolpyruvate within glycolysis. This enzymatic reaction is reversible and depends on the concentration of the substrates available in the environment (Wold and Ballou, 1957a,b). Three isotypes of this enzyme have been described in mammals thus far: α-enolase (ENO-1), ubiquitously expressed in most of the tissues, β-enolase (ENO-3), expressed primary in muscle tissue and γ-enolase (ENO-2), present mostly in neural tissues. All three isotypes form homo-dimers utilizing two magnesium ions non-covalently bound to the active site (Marangos et al., 1978; Brewer, 1981; Pancholi, 2001). In general, glycolytic enzymes are evolutionary highly conserved proteins, exhibiting up to 40–90% amino acid sequence homology among species (Pancholi, 2001). Although most of the glycolytic enzymes could be considered as housekeeping proteins, expression of ENO-1 has been found to dramatically vary depending on the pathological, stress, or metabolic state of the cell. This ENO-1 characteristic has been highlighted in a retrospective study analyzing 169 articles published in the field of proteomics between 2004 and 2006. The authors of this report demonstrated that ENO-1 is the most differentially expressed protein in humans regardless of a tissue type and a pathological condition (Petrak et al., 2008). Consequently, several pathologies with distinct etiologies reported disturbed expression and/or activity of this enzyme. For instance, altered levels of ENO-1 has been demonstrated in Alzheimer’s disease (Castegna et al., 2002; Butterfield and Lange, 2009; Owen et al., 2009), rheumatoid arthritis (Kinloch et al., 2005; Montes et al., 2011), systemic sclerosis (Terrier et al., 2010; Mehra et al., 2013), type 2 diabetes (Li et al., 2013, 2015), systemic lupus erythematosus (Hawro et al., 2015; Li et al., 2018), hepatic fibrosis (Peng et al., 2013; Zhang et al., 2013), and fungal and bacterial infections (Bergmann et al., 2013; Funk et al., 2016; Ji et al., 2016). In addition, ENO-1 has been found to be overexpressed in more than 20 types of human cancer (Altenberg and Greulich, 2004). This finding can be partially explained by the Warburg effect (Vander Heiden et al., 2009), a process, in which cancer cells switch from oxidative phosphorylation to anaerobic glycolysis under hypoxic conditions. However, recent observations have brought interest on the non-catalytic functions of ENO-1 (Pancholi, 2001; Diaz-Ramos et al., 2012; Ji et al., 2016). This glycolytic enzyme (primary localized in the cytoplasm) has been detected at different unexpected sites of the cell, driving such diverse processes as plasminogen binding, maintenance of the mitochondrial membrane stability, RNA chaperone activity and signal transduction. The latter has been found to be associated with the release of ENO-1 to the extracellular space.

The ability of ENO-1 to execute more than one function, depending on its cellular localization, opened the door for ENO-1 to the family of the moonlighting proteins. These unexpected functions of ENO-1 will be discussed in this review.

### ENO-1 Function in Cytoplasm

The primary function of ENO-1 is its catalytic contribution to glycolysis in the cytoplasm. For the detailed biochemical course of this reaction please refer to Qin et al. (2012). Although ENO-1 catalyzes the semi-final step of glycolysis, this enzyme desires more then just an ordinary membership in the long list of glycolytic enzymes. A study by Capello et al. (2016), revealed that silencing of ENO-1 has a dramatic impact on the overall metabolic state of the cell. Proteomic profiling of human pancreatic cancer cells demonstrated that ENO-1 knockdown induces a metabolic shift in these cells. Specifically, ENO-1 silencing promoted β-oxidation of fatty acids, amino acid catabolism, and nucleotide base synthesis. Furthermore, ENO-1 depleted cancer cells evaded the “glycolytic shutoff” by enhancing mitochondrial electron flux followed by increased oxygen consumption, thus restoring oxidative phosphorylation. A functional consequence of these alterations has been observed both, *in vitro* and *in vivo* as ENO-1 depleted cancer cells exhibited decreased growth, survival and clonogenic ability (Capello et al., 2016). Thus, this study highlighs the key role of ENO-1 in the regulation of the Warburg effect in cancer cells. Besides the role of ENO-1 in metabolic shift of cancer cells, ENO-1 may also regulate metabolic processes in other cell types, including pulmonary artery smooth muscle cells (PASMCs) (Dai et al., 2018). Dai et al. (2018), showed that silencing of ENO-1 inhibited hypoxia-induced glycolysis and restored basal respiration and oxygen consumption rate accompanied by increased β-oxidation and glutamine consumption in PASMC. In addition, loss of ENO-1 reduced PASMC proliferation and de-differentiation and induced cell apoptosis in a AMPK-Akt-dependent manner. Consequently, blockage of ENO-1 reversed hypoxia and/or Sugen 5416-induced pulmonary hypertension (PH) in rats. Importantly, these effects of ENO-1 were independent of its catalytic activity as phosphoenolpyruvate, the product of the reaction catalyzed by ENO-1, failed to reverse effects of ENO-1 depletion (Dai et al., 2018).

Furthermore, ENO-1 inhibition turned out to be also beneficial in type 2 diabetes. Type 2 diabetes mellitus is a metabolic disorder characterized by hyperglycemia and insulin resistance. Moreover, it is associated with the obesity and the development of secondary comorbidities, such as liver, kidney, and heart disorders. Interestingly, in an experimental murine model of type 2 diabetes mellitus, inhibition of ENO-1 by the ENOblock reduced blood glucose concentration and LDL cholesterol levels as well as decreased secondary diabetic complications such as adipocyte size, cardiac hypertrophy, expression of inflammatory mediators and attenuated liver fibrosis (Jung et al., 2013; Cho et al., 2017). The ENOblock is a non-substrate cell permeable ENO-1 inhibitor that was originally discovered in a small molecule screening in cancer cell cytotoxicity assays (Jung et al., 2013). These assays help to detect agents that prefentially kill hypoxic cancer cells, thus the cells, which are mainly responsible for the resistance to anti-cancer therapies. Accordingly, ENOblock succesfully decreased migration and invasion of human colon carcinoma cells and inhibited cancer cell dissemination in a zebrafish tumor xenograft model. In addition, ENOblock was found to potentiate the action of the anti-cancer drugs such as taxol and vincristine (Jung et al., 2013).

Cytoplasmic ENO was found to function as a stress-related or as a heat-shock protein. This property of ENO has been described in bacteria, yeasts, parasites, and mammalian cells. For instance, ENO was identified as one of the proteins being overexpressed under acidic conditions in *Streptococcus mutants (S. mutants)*, suggesting that ENO may participate in the survival and proliferation of *S. mutans* in low pH (Wilkins et al., 2002). In *Escherichia coli (E. coli)*, ENO was found to be a major target of stress-inducing oxidative agents (Tamarit et al., 1998), whereas in yeasts, ENO-2 was resported to function as a heat shock protein induced by elevated temperature (Iida and Yahara, 1985). Parasites seem to utilize a similar mechanisms in order to overcome stress conditions. Namely, in *Plasmodium falciparum* (*P. falciparum*), ENO was found to bind to endoprotease (DegP), an enzyme, which plays important role in thermal and oxidative resistance (Sharma et al., 2014). Lastly, findings in cardiomyocytes resemble those obtained in bacteria, yeasts, and parasites. Specifically, the interaction of ENO-1 with heat shock protein 70 was shown to protect cardiomyocytes against oxidative stress (Luo et al., 2011). These results indicate that the stress-related function of ENO is conserved among different species. Besides, ENO-1 was reported to regulate the dynamics of the cytoskeletal filaments. In particular, it has been shown that ENO-1 may interact with tubulin and microtubules during myogenesis (Keller et al., 2007). In addition, ENO-1 can be “activated” upon stress conditions and interact with contractile filaments in cardiomyocytes thus inducing their contraction in a extracellular signal-regulated kinase (ERK) 1/2-dependent manner (Mizukami et al., 2004). Finally, ENO-1 has been detected in the centrosome of HeLa cells during the cell cycle. Although not experimentally proven, it is conceivable, that centrosomal ENO-1 may regulate the interphase cytoskeleton and the mitotic spindle organization (Johnstone et al., 1992).

**Figure 1:**
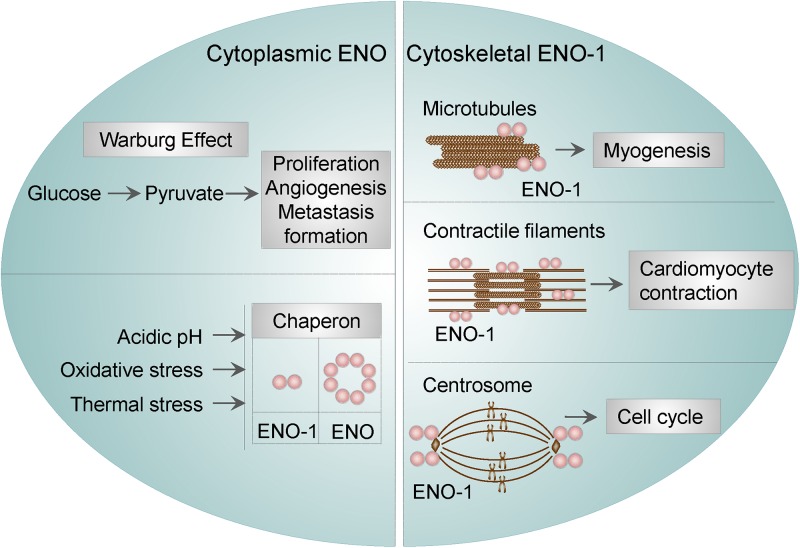
Cytosolic and cytoskeletal functions of ENO-1. Cytosolic ENO-1 is the main enzyme contributing to the glycolysis and its tumor adaptation Warburg effect. In addition, cytoplasmic ENO may function as a chaperone in bacteria and mammalian cells (eukaryotic ENO-1 is presented as a dimer, whereas bacterial ENO is an octamer). Furthermore, ENO-1 can interact with (i) microtubules during the process of myogenesis (ii) contractile filaments of cardiomyocytes upon their contraction and (iii) centrosome during the cell cycle.

Altogether, these findings indicate that, cytosolic ENO-1 irrespective of its catalytic function, contributes to the diverse physiological and pathological conditions (Figure 1).

### Nuclear Activities of ENO

By using an alternative start codon, *ENO-1* gene can give rise to a truncated 37 kDa protein, also called myc promoter-binding protein 1 (MBP-1), which is primary localized in the nucleus. MBP-1 lack’s the first 96 amino acids present in ENO-1 (Subramanian and Miller, 2000) and its primary function is to bind and suppresses the activity of c-myc transcription factor. In detail, MBP-1 recognizes TATA motif in the minor groove of c-myc P2 promoter and negatively regulates its activity by preventing the formation of a transcription initiation complex (Feo et al., 2000; Subramanian and Miller, 2000). The c-myc proto-oncogene is the master regulator of cell proliferation, differentiation and apoptosis (Dang, 2013). Although, both MBP-1 and ENO-1 may bind to c-myc (Feo et al., 2000), only MBP-1 represses activity of this transcription factor and thus acts as a tumor suppressor.

Consequently, MBP-1 expression has been correlated with the clinicopathological features of diverse cancer types including breast, prostate, and gastric cancer. *In vivo* studies demonstrated that, ectopic overexpression of MBP-1 inhibits proliferation, migration, and invasion of cancer cells (Ray et al., 1995; Ghosh et al., 2005a,b; Hsu et al., 2009; Kanda et al., 2009). For instance, Hsu et al. (2009) reported, that MBP-1 suppresses epithelial–mesenchymal transition (EMT) by inhibiting COX-2 expression in gastric cancer. In addition, reduction of cyclin D1 and myocyte-specific enhancer factor 2C (MEF2C) transcriptional activity in prostate cancer cells was noted (Ghosh et al., 2005a). Tumor suppressor activities of MBP-1 have been also observed in different types of human breast carcinomas (Lo Presti et al., 2010). While the majority of breast carcinomas displayed increased ENO-1 levels, the levels of MBP-1 were reduced with a concomitant increased in the activity of c-myc. Loss of MBP-1 expression predicted local recurrence of breast cancer, while its expression correlated with a 92% local recurrence-free survival. No correlation between nuclear MBP-1 expression and cytoplasmic ENO-1 levels was described, suggesting that the expression of MBP-1 is regulated independently from ENO-1 (Lo Presti et al., 2010).

Although the mechanisms regulating MBP-1/ENO-1 ratio are not fully understood, several factors such as hypoxia, ER stress and glucose concentrations were described to influence MBP-1 and ENO-1 transcription. It is well-recognized that sustained hypoxia in growing tumors can enhance the invasiveness of cancer cells by regulating c-myc expression (Huang, 2008). Although *ENO-1* gene gives rise to both, ENO-1 and MBP-1, ENO-1 seems to be preferentially translated upon hypoxia (Sedoris et al., 2010), whereas MBP-1 in response to ER stress (Sedoris et al., 2010; Maranto et al., 2015). Moreover, alterations in the glucose concentration may influence the MBP-1/ENO-1 ratio. Breast cancer cells grown at a physiological or a high concentration of glucose demonstrate increased proliferation with a corresponding decrease in the MBP-1 levels (Sedoris et al., 2007). In contrast, the cells grown at a low concentration of glucose display elevated amounts of MBP-1. In addition to the translational regulation of MBP-1, its interactions with different protein partners may influence its association with the c-myc promoter. For example, the binding of the Kelch protein NS1-BP to MBP-1 was reported to enhance MBP-1-mediated repression of c-myc *in vitro* and *in vivo* (Perconti et al., 2007). Also, Notch 1 receptor intracellular domain (N1IC) was found to associate with MBP-1 as well as with ENO-1. It is known, that N1IC forms a complex with a YY1 transcription factor to activate c-myc. However, binding of both ENO-1 and MBP-1 to N1IC, suppressed N1IC-mediated c-myc transcriptional activity leading to reduced colony-forming ability of human erythroleukemia K562 cells (Hsu et al., 2008). Nuclear activities of ENO have been also detected in plants, in particular, in *Arabidopsis thaliana*. Findings by Lee et al. (2002) demonstrated, that *LOS2*, a genetic locus required for cold-responsive gene transcription, encodes ENO. Los2 protein binds to the promoter of the *STZ/ZAT10* gene, which contains the cold- and osmotic stress-responsive elements and possesses similarities to the MBP1-binding sequence in the human c-myc promoter. Interestingly, Los2 also binds to the DNA probe containing the human MBP-1-binding site in the c-myc promoter, indicating that the plant ENO may function as a transcription factor (Lee et al., 2002).

**Figure 2:**
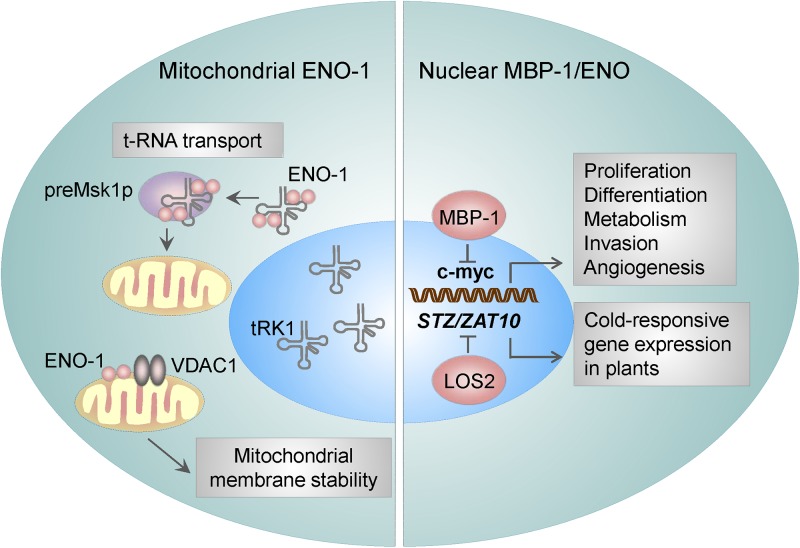
ENO-1 activities in the nucleus and mitochondria. Mitochondria bound ENO-1 acts as a RNA chaperone by binding to and transporting nucleo-cytoplasmic tRNAs to the mitochondria. Furthermore, by binding to the VDAC1 (an integral mitochondrial membrane protein), ENO-1 participates in the stabilization of the mitochondrial membrane potential. By using an alternative start codon, *ENO-1* gene can give rise to truncated 37 kDa protein – myc promoter-binding protein 1 (MBP-1), which suppresses the activity of c-myc transcription factor and thereby regulates proliferation and invasion of cancer cells. ENO in plants is encoded by *LOS2*, which binds to the promoter of the *STZ/ZAT10* gene, that possesses the cold- and osmotic stress-responsive elements.

To sum up, the results mentioned above demonstrate that MBP-1 is a potent tumor-suppressor (Figure 2) and thus a promising target for anti-cancer therapies. Accordingly, a recent study by Cho et al. (2017) reported that, ENOblock induces MBP-1 nuclear localization consequently leading to the decreased expression of c-myc. This resulted in a decreased migration and invasion of human colon carcinoma cells and finally their death (Jung et al., 2013). Future clinical studies will clarify whether this inhibitor may have beneficial effects in cancer or diabetes patients.

### Association of ENO With Mitochondria

Association of ENO with mitochondria was first described in *Arabidopsis thaliana* cells (Giege et al., 2003). Few years later, Entelis et al. (2006) observed a similar effect in yeasts. Namely, ENO-2 and to a lesser extent ENO-1 were found to be tightly bound to the mitochondrial membrane and possessed RNA binding activity (Brandina et al., 2006; Entelis et al., 2006) thus facilitating the import of cytosolic tRNA into mitochondria. As mitochondrial DNA carries information for a small number of macromolecules, there is a constant need for a supply of proteins and RNAs from nucleo-cytoplasmic compartment. In yeasts, two nuclear DNA-encoded tRNAs are targeted to mitochondria: tRK1 (tRNA^Lys^_CUU_) (Martin et al., 1979) and tRNA^Gln^ (Rinehart et al., 2005). The transport of tRK1 is highly specific and requires interaction with the precursor of mitochondrial lysyl-tRNA synthetase (preMsk1p) (Tarassov et al., 1995a,b). However, the interaction of tRK1 with preMsk1p is not sufficient for the transport of tRK1 to mitochondria. Unexpectedly, ENO-2 and ENO-1 have been identified to bind tRK1, enhance tRK1-preMsk1p complex formation and to mediate tRK1 transport to this organelle (Figure 2) (Brandina et al., 2006; Entelis et al., 2006). While the yeast ENO-2 preferentially binds to tRK1, in humans all ENO isoforms may interact with tRK1 and facilitate its transport to mitochondria (Baleva et al., 2015). Interestingly, other glycolytic enzymes, including 3-phospho-glycerate kinase, hexokinase, pyruvate kinase, and glyceraldehyde-3 phosphate dehydrogenase have been found to associate with the mitochondrial membrane too (Brandina et al., 2006). Although the binding of other glycolytic enzymes to mitochondria was found to be relatively weak, it was suggested that they enable direct transport of pyruvate to this organelle.

The functional importance of the above mentioned process has been highlighted during cardiac ischemia and hyperthrophy. Binding of ENO-1 to mitochondria was reported to dramatically decline in cardiomyocytes isolated from the hearts of doxorubicin (Dox)-treated rats (Gao et al., 2014). Doxorubicin is used in the treatment of the breast cancer, however its clinical use is limited as it causes severe side effects like mitochondrial damage in cardiomyocytes and subsequent apoptosis of these cells (Carvalho et al., 2009). Mechanistically, mitochondria isolated from ENO-1 depleted cells display a higher sensitivity to Ca^2+^-induced membrane depolarization and permeabilization and thereby a severe mitochondria dysfunction. This effect can be restored by the addition of the recombinant ENO-1 to the isolated mitochondria, which prevents mitochondrial membrane permeabilization and subsequent organelle fragmentation (Gao et al., 2014). These findings suggest that ENO-1 exerts a protective effect on cardiomyocytes during myocardial injury by stabilizing the mitochondrial membrane potential. Accordingly, further studies revealed that ENO-1 interacts with VDAC1 (Figure 2), an integral membrane protein, which is a critical regulator of mitochondrial membrane stability (Gao et al., 2014).

Although binding of ENO-1 to mitochondria seems to prevent Ca^2+^-induced mitochondrial dysfunction in an isolated system, the concomitant increase of ENO-1 in the cytosol of cardiomyocytes observed upon Dox-treatment of rats was found to be rather harmful. Gao et al. (2015), demonstrated that silencing of ENO-1 in the hearts of rats prevents Dox-induced heart injury and cardiomyocyte apoptosis. This effect was also confirmed in cultured cardiomyocytes and was independent of ENO-1’s catalytic activity. Thus the relationship between ENO-1 and mitochondria in Dox-induced cardiotoxicity is more complex. At one site, association of ENO-1 with mitochondria is protective since it prevents organelle permeabilization in the isolated system. On the other hand, the concomitant increase of the cytosolic ENO-1 after Dox treatment observed *in vivo* is harmful and potentiates Dox-induced heart injury. These contrary results highlight compartment-specific functions of ENO-1.

### ENO in Intracellular Vesicles

**Figure 3:**
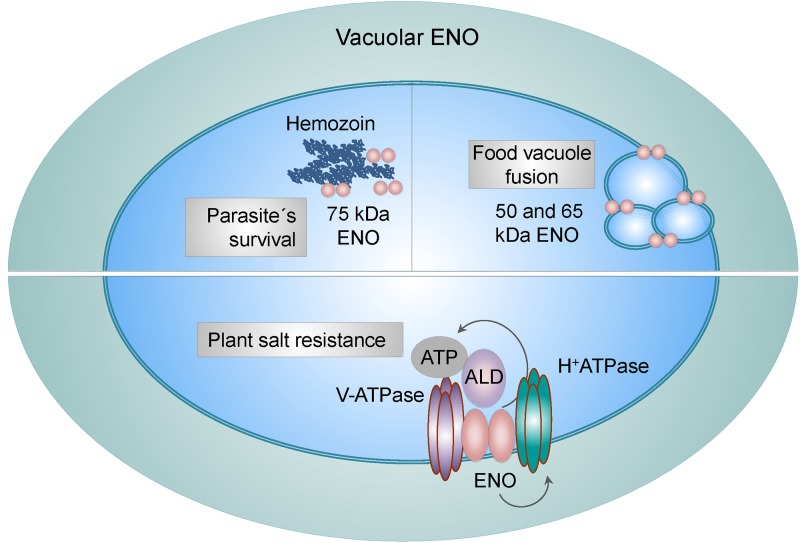
Vacuole-related functions of ENO. Vacuole-associated ENO in parasites exists in three different variants: the low (50 and 65 kDa) and high (75 kDa) molecular weight forms. While the low molecular weight ENO variants regulate fusion of food vacuoles, the 75 kDa form interacts with hemozoin, a disposal product of hemoglobin digestion. Binding of ENO to hemozoin plays a role in parasites survival. In plants, ENO binds to subunits of the vacuolar H^+^-ATPase and V-ATPase and stimulates V-ATPase activity in an aldolase (ALD)-dependent manner by increasing the affinity for ATP. This mechanism is used by plants to overcome high salt concentrations.

Vacuoles present in parasites, yeast and plants are functional equivalents of the mammalian lysosomes. Transport of different molecules between cytosol and vacuole is crucial for many physiological processes including, ion homeostasis, storage of various molecules, detoxification, proteolysis of cytosolic and membrane proteins (Rotin et al., 2000) and turnover of organelles (Kim et al., 2007). Intriguingly, ENO has been recognized as a constituent and as a regulator of vacuoles. For example, in *P. falciparum*, ENO was found to associate with a food vacuole (Bhowmick et al., 2009). Another study identified three different variants of ENO present in this organelle; the typical 50 kDa form and the 65 and 75 kDa forms, the latters were shown to be ubiquitinated. While the low molecular weight forms were reported to assist in the fusion of the food vacuoles, the 75 kDa form of ENO was described to interact with hemozoin, a disposal product formed by the digestion of hemoglobin in blood-feeding parasites (Figure 3). Breakdown of hemoglobin leads to the release of high quantities of free heme, which is toxic to parasites. However, parasites may convert free heme to the non-toxic hemozoin and store it in vacuoles. Neutralization of the toxic heme by parasite specific enzymes is one of the mechanism by which parasites escape host immunity and survive in a hostile environment. Thus, it was suggested that different variants of ENO present in vacuoles may have distinct functional activities (Shevade et al., 2013). Experiments performed in yeasts described similar effects. Proteome profiling of the yeast vacuolar membrane identified ENO-2 to be enriched in this compartment, implying a possible transporter or fusion-related function (Wiederhold et al., 2009). Indeed, a study by Decker and colleagues revealed, that in *Saccharomyces cerevisiae*, ENO-1 and ENO-2 stimulate fusion of vacuoles independently from their catalytic functions (Figure 3). Consequently, deletion of either ENO-1 or ENO-2 caused vacuole fragmentation. Furthermore, ENO-1 or ENO-2 deficiency prevented normal protein sorting to vacuoles, thus exacerbating the fusion defect (Decker and Wickner, 2006). Finally, the association of ENO with vacuoles has been also observed in plants. Barkla et al. (2009) demonstrated that ENO together with aldolase associate with subunits of the vacuolar H^+^-ATPase and V-ATPase and stimulate V-ATPase activity *in vitro* by increasing the affinity for ATP (Figure 3). Consistently, a single point mutation in the 5^′^ region of the *ENO* gene (*los2* mutation), displayed increased sensitivity to salt and an impaired ability to regulate V-ATPase hydrolytic activity in response to aldolase. Furthermore, it was suggested that, ENO not only channels ATP to the V-ATPase, but also directly enhances H^+^-pump’s activity (Barkla et al., 2009). Whether ENO-1 associates with lysosomes in mammalian cells requires further investigations.

### ENO Activities on the Membrane and in Extracellular Space

**Figure 4:**
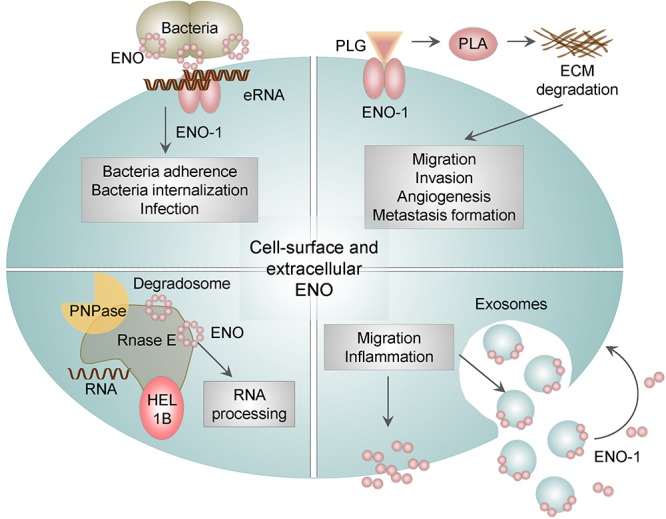
The role of membrane bound and extracellular ENO. In addition to the intracellular activities, ENO-1 may be translocated to the cell surface or released into the extracellular environment as a free soluble protein or bound to exosomes. Surface bound ENO-1 acts as a plasminogen (PLG) binding receptor and participates in plasmin (PLA) formation, which degrades extracellular matrix proteins (ECM). Furthermore, cell surface ENO-1 can bind extracellular RNA (eRNA), which may act as a bridging molecule between bacterial ENO and eukaryotic ENO-1 and thus facilitate bacterial dissemination. In addition, ENO is a part of the bacterial degradosome, which consists of RNase E, RNA helicase 1B (HEL1B) and polynucleotide phosphorylase (PNPase). ENO binding to the degradosome affects the activity of the complex and consequently cellular RNA processing. Lastly, ENO-1 secreted outside of the cell either in the form of exosomes or as a free floating protein may induce migration and a pro-inflammatory phenotype of immune cells.

Next unanticipated location of ENO-1 is cell surface and extracellular space, where ENO-1 either associates with exosomes or is secreted as a soluble protein. Cell surface bound ENO-1 has been detected on several cell types including immune (Miles et al., 1991; Redlitz et al., 1995; Wygrecka et al., 2009), cancer (Didiasova et al., 2014, 2015) and neuronal cells (Haque et al., 2018) as well as bacteria (Pancholi and Fischetti, 1998; Derbise et al., 2004). Outside of the cell, ENO-1 acts as a plasminogen binding receptor (PLG-R) (Figure 4) (Plow and Das, 2009). Plasminogen (PLG) is a zymogen, which is converted to the serine protease plasmin (PLA) in the presence of the physiological activators: urokinase-type PLG activator (uPA) or tissue-type PLG activator (tPA) (Didiasova et al., 2014). Consequently, PLA activates collagenases, degrades fibrin and several other matrix proteins. ENO-1-dependent PLA formation allows pathogens (Chhatwal, 2002) as well as immune (Wygrecka et al., 2009) and cancer cells (Hsiao et al., 2013) to invade tissue, consequently leading to infection, inflammation, and metastasis formation.

We previously demonstrated that pericellular proteolysis mediated by ENO-1 plays a crucial role during the recruitment of monocytes to the inflamed lung. Overexpression studies revealed that ENO-1 potentiated migration and matrix-penetrating capacity of monocytes in a PLA-dependent manner while monocytes bearing truncated ENO-1 variant missing PLG-binding site failed to infiltrate the lung (Wygrecka et al., 2009). Similar effects have been observed in the kidney stone disease, where cell surface bound ENO-1 enhanced migration and invasion of monocytes through renal interstitium (Chiangjong and Thongboonkerd, 2016).

On the cell surface of cancer cells, ENO-1 was reported to regulate migration, invasion and colony formation (Mikuriya et al., 2007; Cappello et al., 2009; Hsiao et al., 2013; Didiasova et al., 2015; Principe et al., 2015). In lung cancer, knock down of ENO-1 decreased the pericellular fibrinolytic activity of cancer cells and thereby their invasion. Consequently, adoptive transfer of an anti-ENO-1 antibody inhibited the formation of tumor metastasis in the lung and the bones (Hsiao et al., 2013). In breast cancer, increased levels of cell surface ENO-1 correlated with enhanced migration and invasion of cancer cells and these effects where abrogated by a specific peptide blocking PLG binding to ENO-1 (Didiasova et al., 2015).

The presence of ENO on the surface of bacteria has been shown to facilitate their migration and tissue invasion leading to the efficient dissemination of bacteria (Figure 3). This mechanism was found to be used by many bacteria including, *S. pneumoniae* (Bergmann et al., 2001, 2013), *Borrelia* spp. (Floden et al., 2011), *Aeromonas hydrophila* (Sha et al., 2009), *Neisseria* spp. (Knaust et al., 2007), *Mycoplasma* spp. (Bao et al., 2014) and by pathogenic fungi such as *Paracoccidioides brasiliensis* (Nogueira et al., 2010), *Aspergillus fumigatus* (Funk et al., 2016), *Candida albicans* (Funk et al., 2016), *Pneumocystis carinii* (Fox and Smulian, 2001) as well as parasites, such as *Leishmania* spp. (Vanegas et al., 2007), *Trypanosoma* spp. (Almeida et al., 2004), and *Plasmodium* spp. (Pal-Bhowmick et al., 2007a,b; Dutta et al., 2015, 2018). Interestingly, we reported that ENO localized on bacterial surface has an additional function to its PLG binding ability. Namely, ENO present on *S. pneumoniae* binds extracellular RNA (eRNA). Extracellular RNA is released from injured cells during infection, it associates on the one hand with ENO present on bacteria and on the other hand with ENO-1 localized on eukaryotic cells thereby facilitating infection of lung epithelial cells. Pretreatment of lung epithelial cells with RNase1 decreases not only the number of adherent bacteria but also the infection of host cells (Zakrzewicz et al., 2016). Taken together our results indicate that upon bacterial infection, eRNA serves as a “bridging molecule” between bacterial ENO and eukaryotic ENO-1 and thereby facilitates pathogen dissemination (Figure 4).

Intriguingly, the membrane-associated ENO in bacteria displays another moonlighting function. Namely, it is involved in the processing of rRNA and degradation of mRNA, and comprises a part of a larger complex termed degradosome. Degradosome is tightly anchored to the inner cytoplasmic membrane of bacteria and is additionally composed of RNA helicase 1B, RNase E, and polynucleotide phosphorylase (PNPase) (Liou et al., 2001). Disruption of the ENO binding to degradosome induces hypoactivity of the complex, which in turn leads to the reduced turnover of cellular RNA (Leroy et al., 2002). In addition, the degradosome-bound ENO was described to play a role in the regulation of the ptsG mRNA stability in *E. coli*. PtsG mRNA encodes the major glucose transporter and is rapidly degraded upon accumulation of glucose-6-P and fructose-6-P in bacteria. Interestingly, deletion of ENO can restore reduced levels of ptsG mRNA, by prolonging ptsG mRNA stability. These results indicate that ENO-mediated regulation of ptsG mRNA stability may coordinate bacterial response to a metabolic stress (Morita et al., 2004).

Despite a large number of studies demonstrating the active role of cell surface bound ENO-1 in invasion of immune, cancer cells and bacteria, the molecular mechanism driving ENO-1 translocation to the cell surface remains unclear. The lack of a signal peptide within the ENO-1 sequence suggests that this protein uses a non-classical secretory pathway to exteriorize. Furthermore, the transport of ENO-1 to the extracellular space was found to be regulated by a plethora of factors. It has been shown that inflammatory stimuli, growth factors, and cell death alter ENO-1 cell surface abundance, thereby increasing the complexity of compartment-specific functions of ENO-1.

Broad range of inflammatory stimuli has been shown to regulate cell surface expression of ENO-1. For instance, LPS potentiated ENO-1 exteriorization in monocytes (Wygrecka et al., 2009) and in cancer cells (Didiasova et al., 2015; Perconti et al., 2017). Stimulation of macrophages with IFN-γ in combination with vitamin D3 (Das et al., 2007) as well as the treatment of cancer cells with CCL-2 or TNF-α (Didiasova et al., 2015) enhanced ENO-1 transport to the cell surface. Dead cells displayed higher PLG binding capacity in comparison to viable cells and this effect was a consequence of enhanced cell surface exposure of different PLG receptors, including ENO-1 (O’Mullane and Baker, 1999; Ucker et al., 2012). Finally, growth factors, like TGF-β1 (Didiasova et al., 2015) and EGF (Perconti et al., 2017) enhanced extracellular transport of ENO-1 in cancer cells.

Though many stimuli have the ability to potentiate cell surface expression of ENO-1, little is known about the mechanisms and intracellular factors that are crucial for the translocation of this glycolytic enzyme from intra- to extracellular space. For example, it was demonstrated that fluctuations in the intracellular levels of calcium, post-translational modifications (PTMs) and interactions with other proteins are crucial for LPS-triggered ENO-1 translocation to the extracellular space. In particular, we showed that the calcium influx is a driving force for ENO-1 transport to the cell surface as well as to the extracellular milieu in the form of exosomes. The stromal interaction molecule (STIM) 1 and the calcium release-activated calcium modulator (ORAI) 1-mediated store-operated calcium entry were found to be crucial for this process (Didiasova et al., 2015). In addition, we observed that exposure of cancer cells to LPS triggers protein arginine methyltransferase 5 (PRMT5)-mediated monomethylation of arginine 50 in ENO-1 (Zakrzewicz et al., 2018) and thus enhances cell surface expression of this protein. Interestingly, several other ENO-1 PTMs, such as ubiqitination, acetylation and phosphorylation were reported too (Pal-Bhowmick et al., 2007b; Foth et al., 2008; Shevade et al., 2013) and ENO-1 ubiquitination was found to direct ENO-1 to vacuoles. In addition, we explored that ENO-1 interaction with caveolin-1 and annexin-2 promotes ENO-1 translocation to specialized subset of lipid rafts called caveolae and thus its transport to the extracellular space (Zakrzewicz et al., 2014). Although it is not clear how these single mechanisms coordinate the process of ENO-1 exteriorization, it is tempting to speculate that the initial increase of intracellular calcium induced by LPS triggers ENO-1 methylation, which works as a sorting signal and marks ENO-1 designated to the cell membrane. Consequently, binding of ENO-1 to caveolin-1 and annexin-2 then facilitates the translocation of this enzyme to the extracellular space. However, the precise molecular mechanism regulating these steps needs to be further investigated.

As indicated above, ENO-1 is not only translocated to the cell surface but also released into the extracellular space either in the form of exosomes (Graner et al., 2009; Henderson and Azorsa, 2012; Didiasova et al., 2015) or as a soluble protein (Guillou et al., 2016). Exosomes are small vesicles that play a role in the intercellular communication and may influence behavior of target cells. We found that exosomes loaded with ENO-1 may enhance cancer cell migration and invasion (Didiasova et al., 2015). Soluble ENO-1 was found in serum and synovial fluid of rheumatoid arthritis patients (Guillou et al., 2016) and in serum of patients suffering from lupus erythematosus (Hawro et al., 2015; Li et al., 2018), systemic sclerosis (Terrier et al., 2010; Mehra et al., 2013) and Alzheimert’s disease (Castegna et al., 2002; Butterfield and Lange, 2009; Owen et al., 2009). In rheumatoid arthritis, ENO-1 was found to reattach to the cell surface of monocytes and stimulate their pro-inflammatory state by inducing the CD14-dependent TLR4 signaling (Guillou et al., 2016). Similar observations have been made in patients with a kidney stone disease. Here, it was proposed that ENO-1 secreted from renal tubular cells may attach not only to monocytes but also to calcium oxalate crystals, the constituents of kidney stones, thus promoting tissue invasion of monocytes and attachment of calcium oxalate crystals to ECM (Chiangjong and Thongboonkerd, 2016).

To sum up, not only surface-bound but also secreted ENO-1 displays strong PLG binding capacity and thus marked impact on local extracellular proteolytic activity. Hence, targeting extracellular ENO-1 may be beneficial not only in the treatment of cancer patients, but also in infectious diseases caused by multiresistant strains and in chronic autoimmune diseases.

## Conclusion and Final Remarks

**Figure 5:**
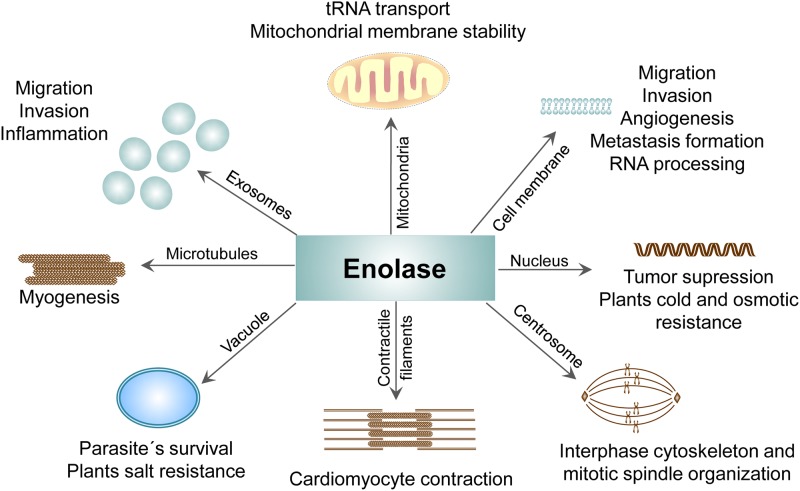
Compartment-specific activities of ENO protein. The presence of ENO in different cellular compartments and its contribution to the physiological as well pathological processes.

The glycolytic enzyme ENO-1 displays highly plastic nature when it comes to its activities. ENO non-glycolytic functions such as cell surface plasminogen binding, maintenance of the mitochondrial membrane stability, transcriptional repressor activity in the nucleus, as well as chaperon and vacuole fusion activity in the cytoplasm imply that the localization of this enzyme determinates its action (Figure 5). Since the mechanistic aspects underlying ENO multitasking remain not understood, future studies are needed to explore the structural and molecular requirements driving ENO shuttling and thus its diverse activities. Nevertheless, the fact that ENO-1 handles high number of cellular processes indicates that ENO-1 targeted therapeutic approaches should be carefully considered. To avoid adverse effects related to the inhibition of the physiological activities of ENO-1 in healthy cells, therapies targeting pathological features of ENO-1 in diseased cells are urgently needed. Nanoparticles with cell-specific targeting may offer such a possibility. A recently published study documented a significant anti-tumor effect of ENO-1 targeted lipid nanoparticles in an animal model of prostate cancer (Wang et al., 2018), thus opening the door for the future clinical trials not only in tumor-related pathologies, but also in other diseases, such as diabetes and cardiac hypertrophy, where ENO-1 related abnormalities are observed as well.

## Author Contributions

MD conceived and wrote the manuscript. LS edited manuscript and provided valuable comments. MW revised the manuscript.

## Conflict of Interest Statement

The authors declare that the research was conducted in the absence of any commercial or financial relationships that could be construed as a potential conflict of interest.
